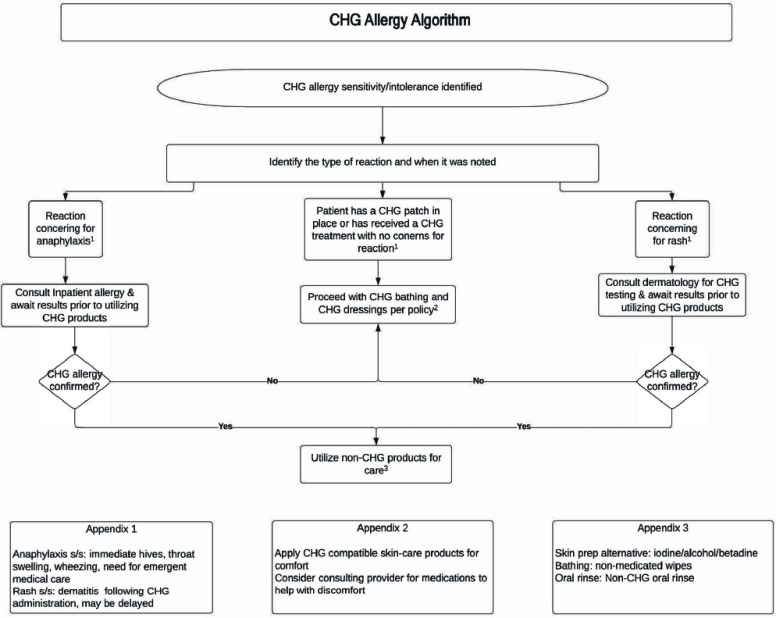# From Label to Enable: Addressing Chlorhexidine Allergies for Safer Patient Care

**DOI:** 10.1017/ash.2025.304

**Published:** 2025-09-24

**Authors:** Erin Gettler, Alexandria Hunt, Shobana Nandakumar, Sabrina Shearer, Walsh Rabina, Seidelman Jessica

**Affiliations:** 1 Division of Infectious Diseases, Department of Medicine, Duke University School of Medicine, Durham, North Carolina; 2 Duke Center for Antimicrobial Stewardship and Infection Prevention, Duke University School of Medicine, Durham, North Carolina; 3 Department of Infection Control and Prevention, Duke University Hospital, Durham, North Carolina; 4 Department of Dermatology, Duke University School of Medicine, Durham, North Carolina

## Abstract

**Background:** Chlorhexidine gluconate (CHG) bathing reduces skin bacterial colonization and reduces the risk of hospital-acquired bloodstream infections, central venous catheter-related bloodstream infections, and transmission of multidrug-resistant organisms. Although mild skin irritation to CHG is common, true IgE-mediated allergy is rare. We implemented a program to delabel low-risk CHG allergies in hospitalized patients. **Methods:** Patients ≥ 18 years of age admitted to inpatient cardiology or hematology-oncology units with an allergy to CHG documented in the electronic medical record were identified. A hospital epidemiologist contacted primary care teams directly, providing a personalized and collaborative approach to evaluate the listed CHG allergy. Using an algorithm devised in partnership with dermatology and allergy experts (Figure 1), guidance was tailored to the reported type of reaction. Each point of contact with care teams was considered an intervention. The primary endpoint was the number of patients for whom a CHG allergy or intolerance was removed from the medical record. **Results:** During a two-month period, 42 interventions for 31 unique patients reporting an allergy to CHG were performed. The cohort was fairly evenly distributed between hematology-oncology and cardiology units (52% vs 48%, respectively), and was 51% male with a median age of 63 (IQR, 20.5 years). Rash was the most frequently listed concern (58%), followed by nonspecific burning or irritation (35%). 5 patients (16%) were delabeled; of these, 2 were directly delabeled due to tolerance of other CHG products (e.g., CHG dressing), and 3 were delabeled by dermatology patch testing. There were no adverse reactions reported after CHG allergy de-labeling. **Conclusion:** Similar to handshake rounds for antimicrobial stewardship, collaborative and targeted education can be an effective strategy to delabel CHG allergies and intolerances.

**Figure:**